# A new fossil cricket of the genus *Proanaxipha* in Miocene amber from the Dominican Republic (Orthoptera, Gryllidae, Pentacentrinae)

**DOI:** 10.3897/zookeys.229.3678

**Published:** 2012-10-22

**Authors:** Sam W. Heads, David Penney, David I. Green

**Affiliations:** 1 Illinois Natural History Survey & Department of Entomology, University of Illinois at Urbana-Champaign, 1816 South Oak Street, Champaign, Illinois 61820, USA; 2 Faculty of Life Sciences, University of Manchester, Manchester M13 9PT, UK; 3 Department of Geology, Amgueddfa Cymru–National Museum Wales, Cathays Park, Cardiff CF10 3NP, UK

**Keywords:** Orthoptera, Grylloidea, Gryllidae, Pentacentrinae, *Proanaxipha*, Dominican amber, Miocene

## Abstract

A new species of the cricket genus *Proanaxipha* Vickery & Poinar (Orthoptera: Gryllidae: Pentacentrinae) from Early Miocene Dominican amber is described and illustrated. *Proanaxipha madgesuttonae* **sp. n.** is distinguished from congeners by: (1) head capsule bearing a distinctive posteriorly bilobed colour spot on the vertex; (2) presence of crossveins in the proximal part of the mediocubital area; (3) apical field of tegmen entirely dark; and (4) median process of epiphallus short. The poorly known *Proanaxipha bicolorata* Vickery & Poinar, of questionable affinity and status, is herein regarded as a *nomen inquirendum*.

## Introduction

The genus *Proanaxipha* was established by Vickery and Poinar (1994) to accommodate two fossil species from Early Miocene Dominican amber; namely *Proanaxipha latoca* Vickery & Poinar, 1994 (the type species) and *Proanaxipha bicolorata* Vickery & Poinar, 1994. Originally placed in the Trigonidiinae, the genus was recently moved to Pentacentrinae by Gorochov (2010) based on close similarities with modern Neotropical pentacentrines. The placement of *Proanaxipha* within Pentacentrinae is well supported by strong dorsoventral flattening of the head capsule, presence of small spines above the metatibial spurs (never present in Trigonidiinae or Nemobiinae) and all veins in the lateral tegminal field running parallel to the costal margin. Moreover, females tentatively identified as *Proanaxipha latoca* by Gorochov (2010) clearly possess ovipositors typical of pentacentrines; i.e. slender and only very slightly curved with a narrow, sharply pointed apex. This differs markedly from the trigonidiine condition, in which the ovipositor is more strongly curved and has a broad, serrated apex. While the inclusion of *Proanaxipha* in Pentacentrinae is beyond doubt, its relationships to other pentacentrine genera have yet to be addressed. In this paper, we describe a new species of *Proanaxipha* from Early Miocene (Burdigalian) Dominican amber and briefly compare the genus with other Neotropical Pentacentrinae.

## Material and methods

The specimen described here is deposited in the Department of Palaeontology, The Natural History Museum, London (NHM) with the accession number NHM II 3048. Photomicrographs were assembled by D.I.G. from a stacked series of digital images captured using a Nikon Coolpix 4500 digital camera mounted on a Leica M10 stereomicroscope with 0.63× and 1.6× planapochromatic objectives. The specimen was studied by S.W.H. using an Olympus SZX12 zoom stereomicroscope and drawings produced with the aid of a *camera lucida*. Morphological terminology generally follows that established by Otte and Alexander (1983) with minor modifications (see Heads 2010a). The age and origin of Dominican amber has been reviewed by Itturalde-Vinent and MacPhee (1996), Grimaldi and Engel (2005) and Penney (2010). The Orthoptera from Dominican amber have been reviewed by Heads (2009, 2010a, b).

## Systematics

### Genus Proanaxipha

Vickery & Poinar, 1994

urn:lsid:orthoptera.speciesfile.org:TaxonName:30586

Proanaxipha Vickery & Poinar, 1994: 15. Type species: *Proanaxipha latoca* Vickery & Poinar, 1994 by original designation – Perez-Gelabert 2001: 72 – Arillo and Ortuño 2005: 8 – Otte and Perez-Gelabert 2009: 142 – Gorochov 2010: 78 [442]. – Penney and Green 2011: 108.

### Proanaxipha madgesuttonae

Heads & Penney sp. n.

urn:lsid:zoobank.org:act:2894AEE9-AD50-48FE-918C-46D86E4F9B0E

urn:lsid:orthoptera.speciesfile.org:TaxonName:76032

http://species-id.net/wiki/Proanaxipha_madgesuttonae

Figs 1 –8

#### Diagnosis.

Distinguished from congeners by the following characters: (1) head capsule with distinctive posteriorly bilobed colour spot on vertex; (2) presence of crossveins in the proximal part of the mediocubital area; (3) apical field of tegmen entirely dark; and (4) median process of epiphallus short.

#### Description.

**Figures 1–2.**
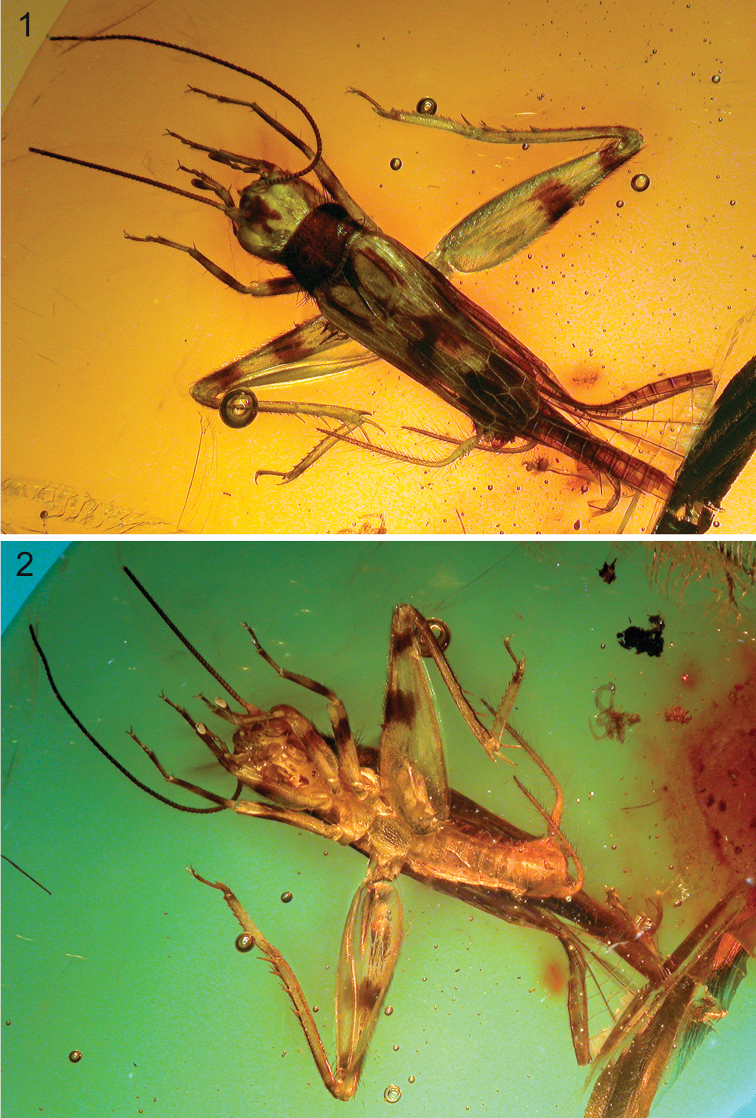
*Proanaxipha madgesuttonae* Heads & Penney, sp. n. Photomicrographs of holotype ♂. **1** dorsal view **2** ventral view.

*Male*: Total body length measured from fastigium verticis to abdominal apex 5.97 mm (Figs 1–2). Head capsule (length 1.26 mm) compressed dorsoventrally; vertex with distinct posteriorly bilobed colour spot (Fig. 4); fastigium verticis broadly rounded; median ocellus situated dorsally, between antennal torulae; compound eyes large, interocular distance 0.64 mm; antennae filiform, scape approximately four times larger than pedicel; maxillary palpi long with apical palpomere triangular and distally concave (see Fig. 2).Pronotum (length 1.02 mm) wider than long, with lateral and marginal areas covered with long setae; disc largely dark with a pale median line not reaching anterior margin; posterior margin sinuous and slightly wider than anterior margin; marginal areas well-demarcated with prominent carinae (Fig. 4). Thoracic sternites polygonal, plate-like and densely pilose, increasing in size posteriorly (Fig. 2). Terminalia obscured dorsally by hind wings (see Fig. 1); subgenital plate pale, broadly rounded with an indistinct median ridge flanked by shallow depressions and the posterior margin shallowly emarginate (Fig. 8); cerci densely setose; epiphallus triangular with pointed apex directed dorsally; ectoparameres lobate.

**Figures 3–8.**
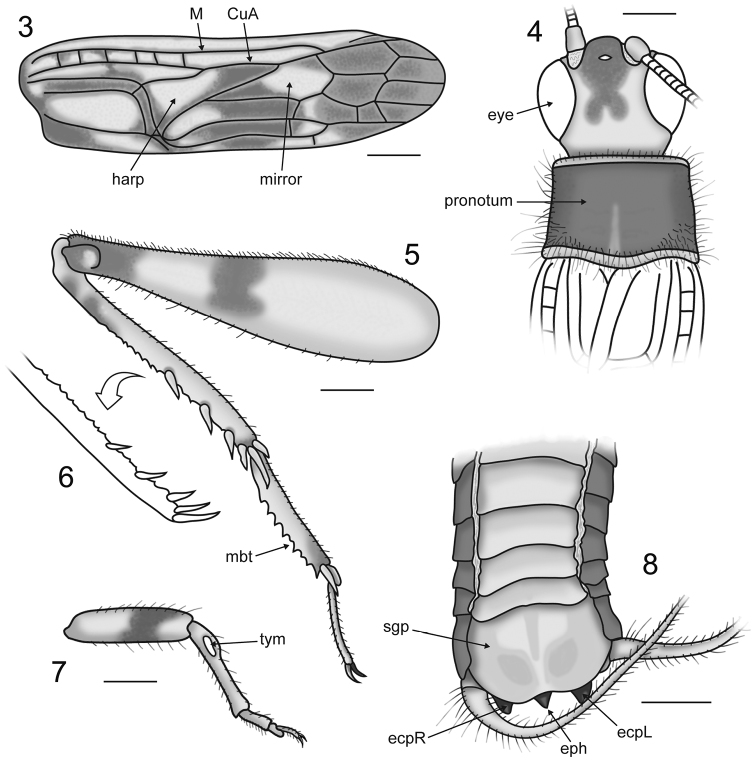
*Proanaxipha madgesuttonae* Heads & Penney, sp. n. Drawings of holotype ♂. **3** dorsal field of right tegmen **4** head capsule and pronotum in dorsal view **5** outer view of right metathoracic leg **6** inner view of right metatibia **7** outer view of right prothoracic leg **8** terminalia in oblique ventral view. Abbreviations: **CuA** anterior cubitus; **ecpL** left ectoparamere; **ecpR** right ectoparamere; **eph** epiphallus; **M** media; **mbt** metabasitarsus; **sgp** subgenital plate; **tym** tympanum. All scale bars 0.5 mm.

Tegmen 3.78 mm long with distinct coloration and stridulatory apparatus only partially reduced (Fig. 3); harp elongate, without multiple harp veins; mirror small, lacking dividing vein; lateral field dark with veins running parallel to the costal margin; dorsal field with six crossveins in the basalmost part of the mediocubital area with dark patches running along the stridulatory and harp veins and merging with a large dark spot encompassing most of the proximal cells in the cubital system; apical field entirely dark (Fig. 3). Hind wing long and tightly folded, extending well beyond abdominal apex, with dark remigium and hyaline anal lobe. Prothoracic leg short and robust with a single dark band on the distal half of the profemur and ovoid tympana on both sides of the protibia (Fig. 7). Mesothoracic leg longer than prothoracic leg; mesofemur with single dark band and a prominent ventral sulcus distally; mesotibiae with two dark bands. Metafemur (length 3.54 mm) with a dense covering of setae and bearing two dark spots; one situated just distad of femoral midlength and the second situated apically, encompassing the genicula (Fig. 5). Metatibia (length 2.61 mm) approximately 25% shorter than metafemur and quadrate in cross section, with two small dark spots situated basally; dorsal longitudinal carinae armed with rows of small denticles interspersed distally with stout subapical spurs (3 inner and 3 outer); metatibial apex bearing 2 inner and 3 outer apical spurs (Figs 5–6); median outer apical spur twice as long as the other outer spurs. Metabasitarsus elongate with rows of sharp denticles along the dorsal longitudinal carinae and two apical spurs (1 inner and 1 outer); second metatarsomere much reduced; third metatarsomere long, slender and slightly curved (Fig. 5).

#### Holotype.

♂: Dominican Republic: Early Miocene (Burdigalian) amber (NHM II 3048).

**Etymology.** Named in honour of Madge Sutton at the request of Dr Susan Shawcross.

#### Remarks.

*Proanaxipha madgesuttonae* is clearly congeneric with the type species *Proanaxipha latoca*, sharing the partially reduced stridulatory apparatus, a long and straight metabasitarsus, similar metatibial armature and the presence of auditory tympana on both faces of the protibia (see Gorochov 2010). Nevertheless, *Proanaxipha madgesuttonae* differs markedly from *Proanaxipha latoca* in its colouration. The new species is altogether darker than *Proanaxipha latoca*, bearing a distinctive posteriorly bilobed colour spot on the vertex of the head capsule (Fig. 4) and a much darker tegmen. The apical field of the tegmen in *Proanaxipha latoca* is either pale or bears a few diffuse dark patches (see Gorochov 2010, p. 443, fig. 6). In contrast, the apical field in *Proanaxipha madgesuttonae* is entirely dark (Fig. 3). The holotype of *Proanaxipha latoca* bears two dark spots on the vertex between the eyes but the rest of the head capsule is pale (see Vickery and Poinar 1994, p. 21, fig. 9). Gorochov (2010) briefly described a number of additional specimens that he tentatively assigned to *Proanaxipha latoca*. However, all of these differ from the holotype in coloration and Gorochov (*op. cit*.,p. 444) remarked that they likely represent a complex of distinct species. While the original colouration of the specimen cannot be known with certainty, arthropod colour patterns are often extremely well preserved in amber (Poinar 1993; Grimaldi and Engel 2005; Penney 2010 and contributions therein). Given the remarkable preservation of the specimen as well as the obvious symmetry of the patterns, it is highly unlikely that they have been altered taphonomically. Morphologically, *Proanaxipha madgesuttonae* is very similar to the holotype of *Proanaxipha latoca* and to the specimens recently described by Gorochov (2010). However, the tegminal venation of *Proanaxipha madgesuttonae* differs in the presence of six crossveins in the basal half of the mediocubital area (Fig. 3). The tegmina are not clearly illustrated in the original description of *Proanaxipha latoca* making it impossible to determine whether or not these crossveins are present. However, the illustrations of *Proanaxipha ?latoca* presented by Gorochov (2010) show no mediocubital crossveins. The distal parts of the phallic complex visible in the holotype of *Proanaxipha madgesuttonae* (Fig. 8) are very similar to those illustrated by Gorochov (2010) though the median process of the epiphallus is shorter in *Proanaxipha madgesuttonae*.

### Proanaxipha bicolorata

Vickery & Poinar, 1994 nomen inquirendum

urn:lsid:orthoptera.speciesfile.org:TaxonName:30588

http://species-id.net/wiki/Proanaxipha_bicolorata

Proanaxipha bicolorata Vickery & Poinar, 1994: 16, fig. 4 – Perez-Gelabert 2001: 72 – Otte and Perez-Gelabert 2009: 142 – Gorochov 2010: 80 [442].

#### Remarks.

Based on Vickery and Poinar’s (1994) photograph of the holotype (a nymph), it is clear that this species does not belong in *Proanaxipha* or even within Pentacentrinae. Unfortunately, neither the illustration nor the original description are sufficient to determine the subfamilial placement of this species, a situation that is further complicated by the nymphal condition of the specimen. Gorochov (2010) suggested a possible affinity with Nemobiinae or Eneopterinae and while both seem possible, neither can be confirmed. Therefore, the species is herein regarded as a *nomen inquirendum* until the type can be redescribed.

## Discussion

The relationships of *Proanaxipha* with other Neotropical Pentacentrinae have yet to be adequately investigated and in the absence of a formal analysis, remain largely unknown. Four extant pentacentrine genera are known from the Neotropical region, namely *Aphemogryllus* Rehn, 1918, *Nemobiopsis* Bolívar, 1890, *Trigonidomimus* Caudell, 1912 and *Velapia* Otte & Perez-Gelabert, 2009. Of these, *Proanaxipha* is most similar to *Nemobiopsis*, sharing with the latter an almost identical arrangement of the metatibial spurs. In particular, Gorochov (2010) drew comparisons between *Proanaxipha* spp. and *Nemobiopsis eugethes* Otte, 2006 from Costa Rica and suggested that the latter species may in fact belong within *Proanaxipha*. *Nemobiopsis eugethes* shares with *Proanaxipha* the presence of tympana on both sides of the protibia, the metabasitarsus elongate and almost completely straight, the male stridulatory apparatus only partially reduced and the somewhat shortened median epiphallic process (Otte 2006; Gorochov 2010). Moreover, while the presence of tympana on both faces of the protibia appears to be unique to *Proanaxipha* and *Nemobiopsis eugethes*,a long and straight metabasitarsus and partially reduced stridulatory apparatus are present in a number of *Nemobiopsis* species (e.g. *Nemobiopsis cavicola* Bonfils, 1981, *Nemobiopsis cortico* Otte & Perez-Gelabert, 2009, *Nemobiopsis decui* Bonfils, 1981, *Nemobiopsis diadromos* Otte & Perez-Gelabert, 2009 and *Nemobiopsis metanasticos* Otte & Perez-Gelabert, 2009). Whether or not these species are more closely allied to *Proanaxipha* than *Nemobiopsis* remains unclear. Indeed, the status of *Nemobiopsis*, *Proanaxipha* and *Grossoxipha* Vickery & Poinar, 1994 (also from Dominican amber) as separate, monophyletic genera is itself questionable and careful revision of the Neotropical Pentacentrinae will undoubtedly shed much-needed light on these problems.

## Supplementary Material

XML Treatment for Proanaxipha

XML Treatment for Proanaxipha madgesuttonae

XML Treatment for Proanaxipha bicolorata
